# Kinking of endotracheal tube during posterior fossa surgery

**DOI:** 10.4103/0019-5049.63629

**Published:** 2010

**Authors:** Neerja Bharti, Indu Bala

**Affiliations:** Department of Anesthesia and Intensive Care, Postgraduate Institute of Medical Education and Research (PGIMER), Chandigarh-160 012, India

Sir,

The airway obstruction during intraoperative period may occur from various causes. It could be difficult to make a diagnosis and secure free airway following airway obstruction due to kinking of endotracheal tube (ETT) in a prone patient with open cranium. We would like to report a case of unexpected ETT kinking in an 8-yr-old male child weighing 22‐kg during posterior fossa surgery in prone position. Anaesthesia was induced with intravenous morphine, propofol and vecuronium. The patient's trachea was intubated orally with a cuffed polyvinyl chloride (PVC) ETT (Portex blue-line, single use) of 5.0 mm. The tube was inserted into the trachea without any resistance and secured at 15 cm at the teeth. Proper ETT position was confirmed by chest auscultation and sustained capnography. Anaesthesia was maintained with propofol infusion and 66% nitrous oxide in oxygen. Patient's lungs were ventilated with volume control ventilation through Datex Ohmeda ventilator using closed circuit. The surgery was started after turning the patient in prone position with flexion of atlanto-axial joint. Toward the end of the surgery (approximately 2.5 h after intubation), the airway pressure began to rise from 24 to 32 cm of H_2_O. Capnography showed normal end-tidal CO_2_ but upward sloping pattern. The oxygen saturation was maintained at ≥97%. There was no sign of bronchospasm on auscultation. No obvious change -in lung compliance was detected on manual ventilation. The nasopharyngeal temperature was 36.4°c at this time. The circuit was checked systematically for kinks, obstructions or leaks but none was found. A 10 F flexible suction catheter was then passed down the lumen of the ETT to exclude partial obstruction. The catheter could not pass down the tube after 6 cm. A palpating finger inside the oral cavity revealed an intraoral kink of the ETT.

**Figure 1:**
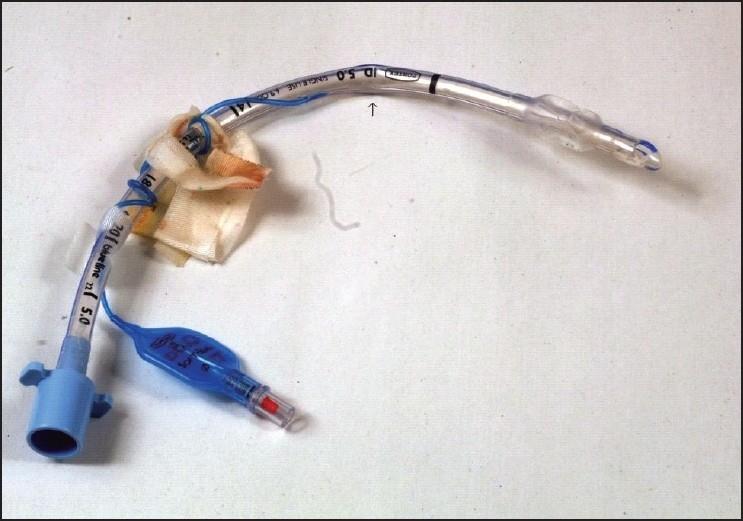
The ETT removed from the patient; arrow indicates the site of kinking

Effective mechanical ventilation and reduction in airway pressure (form 32 to 28 cm of H_2_ O) could be achieved after manual straightening of the kink and repositioning of the head. The surgery was completed without further difficulty. The direct laryngoscopy after turning the patient supine, revealed a partial kink at the oropharynx portion of the ETT. Anaesthesia was reversed and the tracheal extubation proceeded after full recovery of consciousness. Inspection of ETT after it was removed showed partial kinking just beyond the attachment of cuff inflation tube [Figure 1].

The ETT kink during posterior fossa surgery might result from overbending of the softening tube due to oral temperature and neck flexion.[1] Campoy et al. demonstrated higher incidence of kinking of ETT during maxima flexion of atlanto-axial joint.[2] The smaller size tubes may be more prone for airway obstruction. It could be difficult to carry out reintubation in such an awful situation when the patient was prone and in pins with surgery in process. Manual straightening of the tube may be helpful to relive kinking of ETT. In a recent report the placement of Berman intubating airway [3] was found to be helpful in these situations. Emphasis should also be laid on the proper positioning of the head and neck prior to surgery. The use of reinforced, nonkinking ETT may be considered in high-risk patients.
